# Reliable Polymerase Chain Reaction Methods for Screening for Porcine Endogenous Retroviruses-C (PERV-C) in Pigs

**DOI:** 10.3390/v17020164

**Published:** 2025-01-24

**Authors:** Hina Jhelum, Dusan Kunec, Vasileios Papatsiros, Benedikt B. Kaufer, Joachim Denner

**Affiliations:** 1 Institute of Virology, Free University Berlin, 14163 Berlin, Germany; hina.jhelum@fu-berlin.de (H.J.); dusan.kunec@fu-berlin.de (D.K.); benedikt.kaufer@fu-berlin.de (B.B.K.); 2 Faculty of Veterinary Medicine, Clinic of Medicine (Farm Animal Medicine), University of Thessaly, GR 43100 Karditsa, Greece; vpapatsiros@vet.uth.gr

**Keywords:** porcine endogenous retroviruses (PERVs), PERV-C, polymerase chain reactions (PCR) methods, real-time PCR, xenotransplantation, porcine viruses, Auckland Island pigs

## Abstract

Porcine endogenous retrovirus C (PERV-C) is a gammaretrovirus present in the genome of many, but not all, pigs. It is an ecotropic virus, able to infect only pig cells. In contrast, PERV-A and PERV-B, which are present in all pigs, can infect cells of multiple host species, including humans, thereby posing a risk for xenotransplantation when pigs are used as donor animals. Notably, PERV-C can recombine with PERV-A to produce PERV-A/C recombinants that can infect human cells and replicate to higher titers compared to the paternal PERV-A. The objective of this study is to evaluate the reliability of both existing and newly developed polymerase chain reactions (PCR) methods for detecting PERV-C, with the aim of selecting PERV-C-free pigs to be used for xenotransplantation. To detect PERV-C by PCR, specific primers targeting the region of the envelope protein gene, which differs from that of PERV-A and PERV-B due to its unique receptor binding site, must be employed. In this study, new PCR assays were developed to detect PERV-C and a total of ten PCR assays and one real-time PCR assay were evaluated for their reliability in detecting PERV-C. These assays were used to screen indigenous Greek black pigs, Auckland Island pigs, and German slaughterhouse pigs. Two of the PCR assays consistently yielded reliable results, whereas the other PCRs and the real-time PCR gave false positive results. Using the reliable assays, it was shown that one out of four indigenous Greek black pigs (using the same method in a previous publication 11 of 21 pigs were found PERV-C-negative), one out of ten German slaughterhouse pigs, the pig kidney cell line PK15, and all the Auckland Island pigs were PERV-C-negative. The reliable PCR assays will enable the screening of PERV-C-negative donor pigs to be used in xenotransplantation. Most importantly, all the Auckland Island pigs that were genetically modified in Germany for use in clinical trials were PERV-C-negative.

## 1. Introduction

Porcine endogenous retroviruses (PERVs) are integrated in the genome of all pigs. PERV-A and PERV-B are present in all pigs, whereas PERV-C is present in not all, but most, pigs [1]. This correlates with the fact that PERV-C is phylogenetically younger than PERV-A and PERV-B [2,3,4]. Since PERV-A and PERV-B can infect human cells (human-tropic viruses), they pose a direct risk for xenotransplantation using pig cells, tissues, or organs [5]. PERV-A and PERV-B infect also cells from numerous other species (polytropic viruses). PERV-C infects only pig cells (ecotropic virus) and, therefore, theoretically, does not pose a risk for xenotransplantation. However, PERV-C can recombine with PERV-A, acquiring the receptor binding site for the receptor on cells of humans and other species, enabling the virus to efficiently replicate in human cells [6,7]. In addition, the replication rate of the PERV-A/C recombinants is higher compared to that of paternal PERV-A [8,9].

Therefore, it is recommended to use PERV-C-free animals as donor pigs for xenotransplantation [10]. To ensure that animals are PERV-C-free, sensitive and specific conventional and real-time PCRs have been developed [1,11,12,13,14,15]. Since the polymerase gene (*pol*) and the gene encoding the core protein (*gag*) are highly conserved among PERV-A, PERV-B, and PERV-C, PERV-C-specific primers must target sequences in the envelope (*env*) gene, which differ between these viruses. These differences correspond mainly to the receptor-binding sites that are specific to each virus.

It is well known that PERVs are active in living pigs [16]. This is demonstrated by the different copy numbers of integrated proviruses in different organs and in different parts of one organ and by an increase in the copy number with age [17,18,19,20,21]. The lowest level of PERV-A and PERV-B proviral DNA has been shown in muscles [20]. These investigations were performed using primer and probes binding to a highly conserved region in PERV’s polymerase sequence (pol), not discriminating between PERV-A, PERV-B, and PERV-C. Furthermore, there is good evidence that PERV-C is active in living pigs: recombinants with PERV-A were found in somatic cells but PERV-A/C recombinants have never been detected in the germ line [6,7].

When we screened indigenous Greek black pigs for PERV-C using a PCR assay, designated PCR4 [12], 11 of 21 animals were found positive [22]. None of the PERV-C-positive animals were positive for PERV-A/C. Indigenous Greek black pigs live in mountainous areas, and they are resistant to weather conditions and to diseases even though numerous viruses including porcine cytomegalovirus/porcine roseolovirus (PCMV/PRV), porcine lymphotropic herpes virus-3 (PLHV-3), and porcine circovirus 3 (PCV3) have been found in most of these animals [22].

Auckland Island pigs are ideal organ donors for xenotransplantation because of their optimal organ size for humans [23]. Therefore, there is no need for a knock-out of the porcine growth factor receptor as is performed with other pig breeds [24]. The second important advantage is the low number of porcine microorganisms. These animals have lived for a hundred years on the remote Auckland Island; they were thoroughly studied in New Zealand [25,26,27] and were used as donors of pig islets in the first clinical trials for treating diabetes patients in New Zealand and Argentina. In these trials, no transmission of porcine viruses, including PERV, to the patients was observed [28,29]. Since more than 50% of the animals in New Zealand have been found to be PERV-C-positive [30], we selected PERV-C-negative animals, cells from which were delivered to Germany, and from these, piglets were obtained by somatic cell nuclear transfer (SCNT) [21]. The genetic diversity, growth, and heart function of these Auckland Island pigs at the Center for Innovative Medical Models (CiMM) in Munich as well as the absence of PERV-C demonstrate that they are excellent donor animals [23].

One objective of this study was to develop new PCR assays for the detection of PERV-C and to evaluate the reliability of both existing and newly developed PCR methods for detecting PERV-C, with the aim of selecting PERV-C-free pigs to be used for xenotransplantation. We used these methods to analyze indigenous Greek black pigs, Auckland Island pigs, and recently analyzed German slaughterhouse pigs [31], as well as a well-characterized pig cell line. The primary objective was to ensure that the Auckland Island pigs, which will be used for clinical trials in Germany, were PERV-C-negative.

## 2. Materials and Methods

### 2.1. Animals and Tissues

The pig materials analyzed and their characterization are shown in Table 1.

### 2.2. DNA Isolation and Characterization

DNA was isolated from the tissues according to the manufacturer’s instructions using the DNeasy Blood & Tissue kit (Qiagen, Hilden, Germany). DNA concentrations were determined using NanoDrop ND-1000 (Thermo Fisher Scientific Inc., Worcester, MA, USA) or Qubit (Invitrogen, Thermo Fisher Scientific Inc., Worcester, MA, USA). To characterize the quality of the DNA, the optical density ratio at 280/260 was determined and documented. The 260/230 ratio was used as a secondary measure of nucleic acid purity. The 260/230 values were in the range of 2.0–2.2. Since we performed a duplex real-time assay estimating both PERV-C and cellular GAPDH, we used GAPDH as an internal process control (IPC). The Ct values of GAPDH in all samples were nearly identical, indicating that DNA isolation was consistent across all probes and that the PCR reaction for GAPDH was also uniform for all probes.

### 2.3. PCR and Real-Time PCR

Conventional PCR was performed to determine the presence of PERV-C using a set of primers (Table 2) [1,12,15,33]. Some of the reactions were carried out with DreamTaq DNA polymerase (Thermo Fisher Scientific, Worcester, MA, USA) and the others were performed with high-fidelity HiFi polymerase (PCR Biosystems, London, UK). All reactions were set up with a Biometra TRIO cycler (Analytik Jena, Jena, Germany).

Each sample was subjected to an initial denaturation for 10 min at 95 °C (one min for HiFi polymerase), followed by 45 amplification cycles of 95 °C for 15 s (35 cycles for HiFi polymerase); the annealing temperatures are shown below (30 s for DreamTaq and 15 s for HiFi Polymerase). The corresponding extension times at 72 °C for each PCR are shown below. In the case of DreamTaq DNA polymerase, but not HiFi polymerase, a final extension at 72 °C for 5 min was added. The annealing temperature for PCR1 was 55 °C; for PCR4, it was 60 °C; for PCR5, PCR6, PCR7, and PCR8, it was 58 °C; for PCR9, it was 55 °C; and for PCR10, it was 58 °C. The extension times used were 9 s for PCR1 and PCR4, 10 s for PCR5, 16 s for PCR6, 5 s for PCR7, 21 s for PCR8, 9 s for PCR9, and 24 s for PCR10 using HiFi polymerase. As positive control, lung tissue from a PERV-C-positive pig that was used as donor pig for heart transplantation into baboon N was used [34]. Water was used as negative control.

In addition, a real-time PCR was established using specific primers and probes (Table 2) [12,15]. A mass of 100 ng DNA and the SensiFAST Probe No-ROX kit (Meridian Bioscience, Newtown, OH, USA) in a 20 µL reaction volume was used. The cycling conditions used were as follows: initial denaturation for 5 min at 95 °C, followed by 45 amplification cycles at 95 °C for 15 s, annealing at 58 °C for 30 s, and extension at 72 °C for 30 s in a qTOWER3 G qPCR cycler (Analytik Jena, Jena, Germany). A standard curve was produced using the 510 bp amplicon of previously described PCR6 [15] as a template. This PCR was performed as follows: 100 ng of DNA template, PCR buffer I containing MgCl2, 0.2 mM dNTPs, and one unit of DreamTaq DNA polymerase (Thermo Fisher Scientific, Worcester, MA, USA). In case of PCRBIO HiFi polymerase, PCRBIO reaction buffer along with one unit of HiFi polymerase was used. As positive control, DNA from the PERV-C-positive donor pig described above was used, and water was used as negative control. The sensitivity of the real-time PCR was 10 copies/100ng DNA [22].

### 2.4. Sanger Sequencing

PCR8 amplicons from pigs 2 and 4 were gel-purified using a DNA gel extraction kit (Monarch, New England Biolabs, Frankfurt am Main, Germany) and sent for sequencing (LGC Genomics, Berlin, Germany). Sequences were analyzed using ApE v3.1.4 software.

### 2.5. Nanopore Sequencing

PCR8 amplicons from pig 1, 3, and 4 were gel-purified using DNA gel extraction kit (Monarch, New England Biolabs) and sent for sequencing (Plasmidsaurus, Eugene, OR, USA). Sequences were analyzed using SnapGene (San Diego, CA, USA).

### 2.6. Sequence Alignment and Phylogenetic Tree

Sequence alignment and phylogenetic tree generation were carried out using Clustal Omega using sequences obtained from nanopore sequencing. ApE plasmid editor was used to align the sequences obtained from Sanger sequencing. The PERV-C reference genome (accession number AM229312 [33]) and the PERV-A reference genome (accession number AY288779.1 [35]) were used for alignment.

**Table 2:** Primers (fw, forward; rev, reverse) and probes used for the detection of PERV sequences (location according to Accession Nr. AM229312) and porcine GAPDH (pGAPDH).

Primer/Probe	Sequence 5′-3′	Location ^a^	PERV-C ^b^	PERV-A ^c^
PERV-C				
PCR1 fwd ^d^	CTGACCTGGATTAGAACTGG	6606-6625	yes	no
PCR1 rev ^e^	CCAGGACCATCCTCTAACAT	6867-6886	yes	no
PCR4 fwd	GATTAGAACTGGAAGCCCCAAGTGCTCT	6614-6641	yes	no
PCR4 rev	ACCATCCTCTAACATAACTTCTGGATCAGA	6872-6901	yes	no
PCR5 fwd	CTATTCGCCTCAAAATAAACCAG	6778-6800	yes	no
PCR5 rev = PCR8 rev = PCR9 rev	CATAGAGACCAATGCACATG	7086-7105	yes	no
PCR6 fwd = PCR8 fwd = PCR10 fwd	CCAGGACCACCAAATAATGG	6435-6454	yes	no
PCR6 rev = envC real time rev	ACTAAAATGGGGGCAAAACTT	6924-6944	yes	no
envC real-time fwd = PCR9 fwd	CCCCAACCCAAGGACCAG	6853-6870	yes	no
envC real-time probe	FAM-CTCTAACATAACTTCTGGATCAGACCC-BHQ1	6878-6904	Yes	no
PCR10 rev	CACTGAAGCCTTTAATCAAACC	7183-7205	yes	no
PERV-A/C (380 bp amplicon)				
PERV-A-VRB ^f^ fwd	CCTACCAGTTATAATCAATTTAATTATGGC	6129-6158	no	yes
PERV-C rev	TATGTTAGAGGATGGTCCTGGTC	6451-6473	yes	no
PERV-A/C (1260 bp amplicon)				
PERV-A-VRB—fwd PERV-C-TMR ^g^ rev	CCTACCAGTTATAATCAATTTAATTATGGC CTCAAACCACCCTTGAGTAGTTTCC	6129-6158 7370-7395	no yes	yes no
pGAPDH				
pGAPDH-fwd pGAPDH-rev pGAPDH-probe	ACATGGCCTCCAAGGAGTAAGA TCAGTGTCGGGGTTGAGCTAG HEX-CCA CCA ACC CCA GCA AGA G-BHQ			

^a^ Location of the PERV-C primer/probe according to accession number AM229312 [33], of the GAPDH primer/probe according to Duvigneau et al., 2005 [36], and primers to detect PERV-A/C as described by Wood et al. [7]; ^b^ primer/probe sequence is present in PERV-C sequence with accession number AM229312, clone1312 [33]; ^c^ primer /probe is present in the PERV-A sequence with accession number AY288779.1 [35]; ^d^ fwd, forward primer; ^e^ rev, reverse primer; ^f^ VRB, variable region B; ^g^ TMR, transmembrane region.

## 3. Results

### 3.1. PCR Methods Used for the Detection of PERV-C

**Figure 1:**
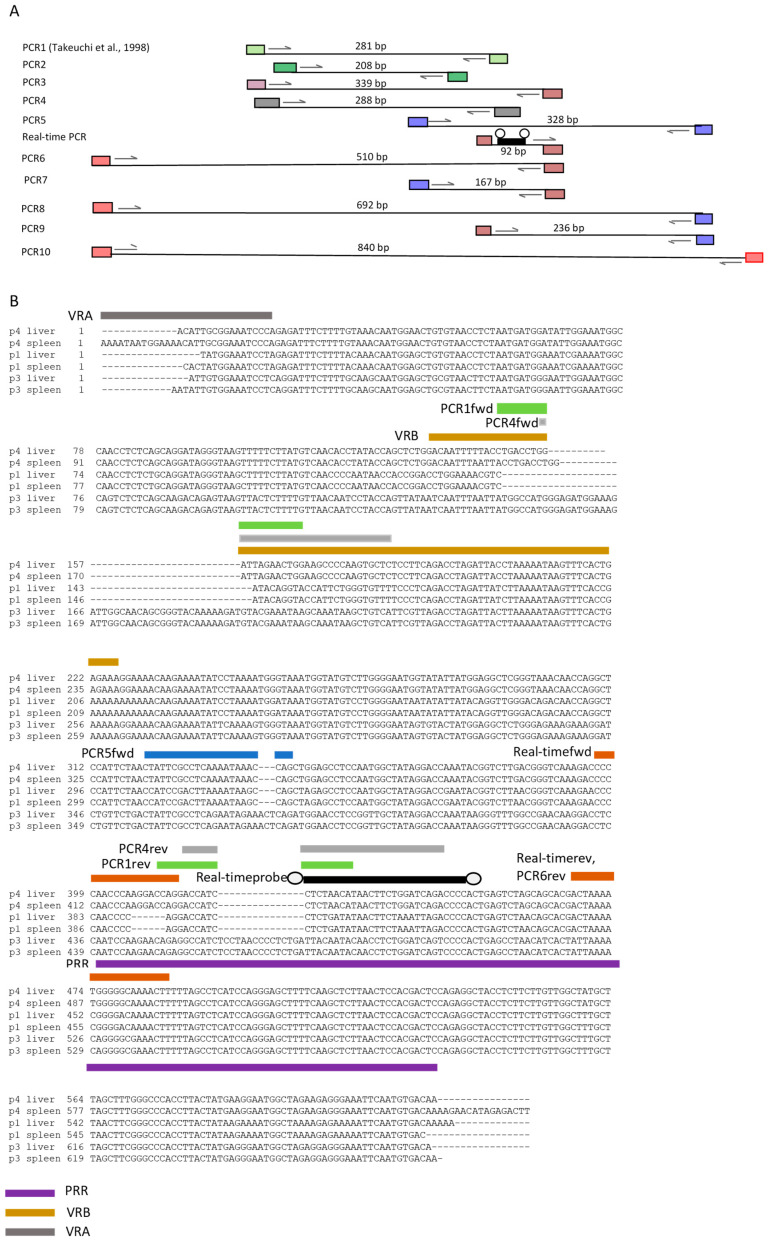
(**A**), Schematic presentation of the used PCRs, the localization of the primer binding sites, and of the probe binding site in the envelope, *env*, sequence of PERV. The lengths of the amplicons are shown. PCR1 was developed by Takeuchi et al. [1]. bp, base pair. (**B**) Results of the nanopore sequencing of amplicons from the PCR8 of DNA from spleen and liver of Greek animals 1, 3, and 4. The primer binding sites and the probe of the different PCR methods are indicated. P, pig; fwd, forward primer; rev, reverse primer. Regions of the receptor binding site in the *env* sequence: VRA, variable region A; VRB, variable region B; PRR, proline-rich region. The circles at the beginning and the end of the probe used for the real-time PCR represent HEX (hexachlorofluorescein) and BHQ (black hole quencher), respectively.

A total of ten PCR assays and one real-time PCR assay, either existing or newly developed, were used to screen for PERV-C. PCR1 is a PCR developed by Takeuchi et al. [1]; and PCR2, PCR3, PCR4, PCR5, and the real-time PCR were developed by Kaulitz et al. [12]. PCR4 was originally designed by Dieckhoff et al. [32], using primers based on a PERV-C sequence [35] (Figure 1A). All primers corresponded to the specific location of the PERV-C receptor binding site in the envelope, *env*, gene of PERV (Figure S1). The primers were located between variable region A (VRA), variable region B (VRB), and proline-rich region (PRR) of the receptor binding site (Figure 1). PCR1 detected up to 1.1 × 10^3^ molecules of a PERV-C plasmid, and the real-time PCR performed as a duplex PCR simultaneously detecting PERV-C and porcine cyclophilin detected 100 copies/reaction of PERV-C [12]. PCR1, PCR2, and PCR3 detected PERV-C equally well in German landrace pigs [12]. In a later publication, we described two new PCRs, PCR6 and PCR7 [15]. All the primer and probe sequences were found in the PERV-C reference sequence (accession number AM229312, [33]), but not in the PERV-A reference sequence (accession number AY288779.1 [35]) (Table 2). The real-time PCR was originally performed as a duplex real-time PCR detecting the housekeeping gene cyclophilin [12]. Here, porcine GAPDH was used (Table 1); the sensitivity was 10 copies of PERV-C/100ng DNA [21]. Furthermore, three new PCRs, PCR8, PCR9, and PCR10, were added, which partially were designed to obtain larger amplicons for sequencing purposes (Figure 1A). All the primer and probe sequences were found in our PERV-C reference sequence, but not in the PERV-A reference sequence (Table 2). Table S1 provides an overview of the PCR and real-time PCR analyses conducted, highlighting that the majority of data identifying PVR1 and PCR4 as the most reliable methods were derived from testing indigenous Greek black pigs.

### 3.2. Application of These Methods to Detect PERV-C in Different Pigs

**Figure 2:**
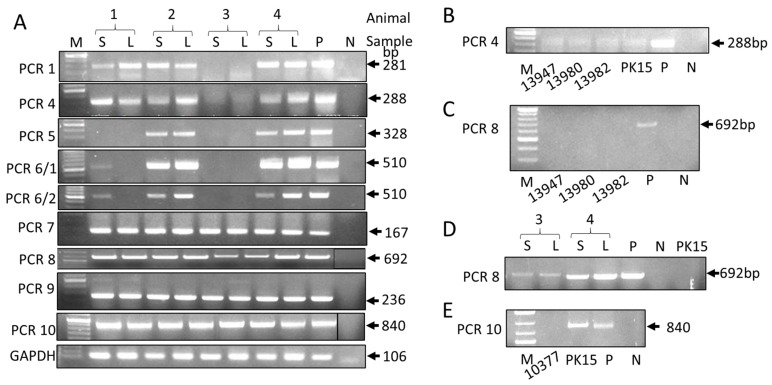
(**A**) Results of different PCRs using DNA from liver and spleen from 4 indigenous Greek black pigs (animals 1, 2, 3, and 4) from farm 1. GAPDH was used to demonstrate identical DNA loading. (**B**) Results of PCR4 and (**C**) of PCR8 using DNA from three Auckland Island pigs (13947, 13980, and 13982) and from PK15 cells. (**D**) Results of PCR8 using DNA from spleen and liver of Greek black pigs 3 and 4 and PK15 cells. (**E**) Results of PCR10 using DNA from the Auckland Island pig 10377 and PK15 cells. S, spleen; L, liver; P, positive control (lung tissue from a PERV-C-positive animal); N, negative control (water); M, marker; bp, base pair.

To evaluate the specificity of the developed PCRs and to determine the presence or absence of PERV-C, we screened indigenous Greek black pigs, Auckland Island pigs, and pigs from a German slaughterhouse. Previously, 21 indigenous Greek black pigs were screened for PERV-C using PCR4. This screening revealed that 11 of 21 animals (52.4%) were positive [22]. To further investigate the presence of PERV-C in these animals, four animals from farm 1 were analyzed using PCR1. Screening with PCR1 reconfirmed the presence of PERV-C in all pigs, except pig 3 (Figure 2A). The same result had been obtained for PCR4 [22]. The absence of PERV-C-specific bands in PCR1 and PCR4 for animal 3 suggests that PERV-C is either absent in this animal or that the primer-binding sites are mutated, making them unrecognizable by one or both primers.

To test these possibilities, PCR5 and PCR6 were performed (Figure 2A). Testing the DNA from the liver and spleen of animals 2 and 4 with PCR5 and PCR6 produced strong amplicons identical in size to that of the positive control. No amplification was observed with PCR 5 and PCR6 when DNA from both tissues of animal 3 was used. Interestingly, no amplification was detected in animal 1 with PCR 5, and only a faint band was observed with PCR6 using DNA isolated from the spleen, but not from the liver. Given the importance of this PCR6 result, the PCR was repeated several times to ensure reproducibility, and the results of the two experiments are presented in Figure 2A. The difference between the liver and spleen suggests that there is a mutation in one or both primer binding sites in the provirus in the liver, but not in the spleen, indicating different proviruses in these organs.

To obtain a larger amplicon for sequencing purposes, PCR8 was performed, using the forward primer of PCR6 and the reverse primer of PCR5. To our surprise, DNA extracted from all the tissues from all four animals yielded amplicons, including Greek pig 3. Most interestingly, the amplicons from the liver and spleen of animal 3 were larger compared to the amplicons from animals 1, 2, and 4, indicating a potential insertion (Figure 2A). PCR7 (the combination of the forward primer of PCR5 and the reverse primer of the real-time PCR) and PCR9 (using the forward primer of the real-time PCR and the reverse primer of PCR5) were also positive for all four animals. When the DNA from the German slaughterhouse pigs was screened using a real-time PCR, all the animals were also positive (Table 3). In contrast, the Auckland Island pigs and the PK15 cells were negative in the real-time PCR (Table 3). The pig cell line PK15 [37] is well known to be PERV-C-negative. The Auckland Island pigs and the PK15 cells were also PERV-C-negative in this study when PCR4 (Figure 2B) and PCR8 were performed. PCR8 used the forward primer of PCR6 and the reverse primer of PCR5 in order to obtain extended amplicons (Figure 2C,D).

### 3.3. Sequence Analysis of PERV-C from Indigenous Greek Black Pigs

The Sanger sequencing of the PCR8 amplicon from Greek pig 4 resulted in a high-quality sequence and an alignment of this sequence with a PERV-C reference sequence AM229312 [33] revealed that the sequence from Greek pig 4 had only a few mutations in the forward primer binding site and no mutations in the reverse primer binding site (Figure S2).

However, since the Sanger sequencing of the other amplicons did not produce satisfactory results, possibly due to the simultaneous amplification of different proviruses present in the pig genome with differences in the sequence, all the amplicons were sequenced using the nanopore method. As expected, putative insertions were detected in the sequence from animal 3 when compared with the sequences from animals 2 and 4 (Figure 1B). The largest insertion disrupts the binding sites of the forward primers of PCR1 and PCR4, while the other disrupts the binding sites of the reverse primers of PCR1 and PCR4. The smallest insertion disrupts the binding site for the forward primer of PCR5. Consequently, none of these three PCRs produced amplicons when DNA from animal 3 was tested (Figure 2A). However, when the large insert and the whole-amplicon sequence from animal 3 were analyzed using nucleotide BLAST (nBLAST), high sequence similarity with PERV-A was observed (Figure S3), indicating that the provirus amplified with PCR8 in Greek pig 3 is PERV-A.

**Figure 3:**
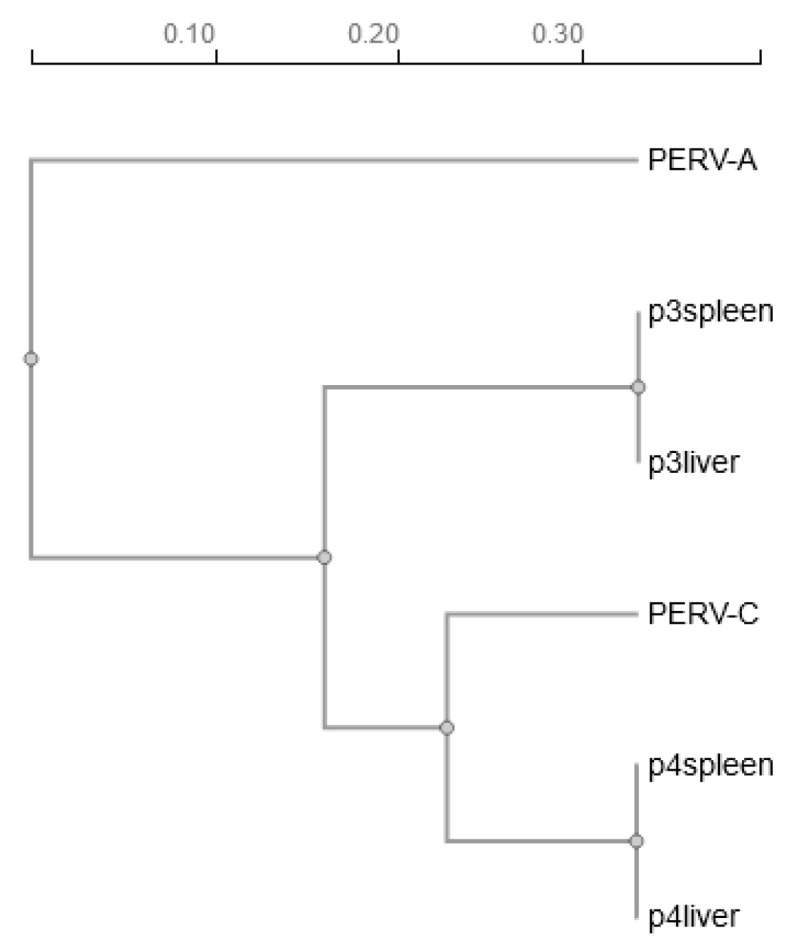
Phylogenetic tree of the nanopore sequences of the amplicons of PCR8 from indigenous Greek black pig 3 and 4 in comparison with the PERV-C reference genome (accession number AM229312 [33]) and the PERV-A reference genome (accession number AY288779.1 [35]). Scale bar refers to a phylogenetic distance of 0.05 nucleotide substitutions per site.

Based on the nanopore sequences of the PCR8 amplicons from animals 3 and 4, a dendrogram was built, showing that the amplicon from animals 4 is closer to the PERV-C reference sequence and the amplicon from animal 3 is closer to the PERV-A reference sequence (Figure 3).

### 3.4. Analysis of the Primer Binding Sites

The sequences obtained by nanopore sequencing of the 692bp amplicons obtained by PCR8 were used to analyze the primer binding sites of PCR1, PCR4, PCR5, PCR6, PCR7, PCR8, and PCR 9, as well as the binding sites for the primers and probes of the real-time PCR (Table 4). The nanopore sequences were of high quality and the sequences of the amplicons derived from the liver DNA and the spleen DNA were 100% identical. When the sequences of the primers and the corresponding sequences in the amplicons were compared, it was confirmed that the forward and reverse primers of PCR 1 and PCR4 are highly specific, because in the PERV-A sequence (amplicon of PCR8 from animal 4), the primer binding sites were disrupted by larger sequences absent in PERV-C. The sequences of the PCR5 primer binding sites in Greek pig 3 had mutations; in the case of the forward primer, an insertion of sequence “AAC” disrupted the primer binding site (Table 4), explaining the absence of amplicons when PCR5 was performed (Figure 2A).

### 3.5. The Real-Time PCR Is Not PERV-C-Specific

In addition, a real-time PCR, as described by Kaulitz et al. [12], was applied. The primers and probe had been designed using sequences found in the PERV-C reference genome, but not in the PERV-A reference genome (Table 2). An identical copy number in the spleen and liver of animals 2 and 4 (21 Ct) was found, but a lower copy number was detected in both tissues of animals 1 and 3 (26 Ct) (Table 3). Interestingly, no significant differences in the PERV copy numbers were observed between the liver and spleen of any the animals. When the real-time PCR was used to screen the four indigenous Greek black pigs from farm 1, all four animals, including pig 3, which was found to be PERV-C-negative on the basis of PCR1, PCR4, PCR5, and PCR6 (Figure 2A), were found to be positive according to the real-time PCR (Table 2). In contrast, the Auckland Island pigs, which were also found to be PERV-C-negative using the mentioned PCRs, were negative according to the real-time PCR (Table 3). All ten German slaughterhouse pigs were positive on the basis of the real-time PCR, including pig 3, which was found to be PERV-C-negative using PCR1 and PCR4 [31] (Table 3). Therefore, the real-time PCR used was not PERV-C-specific.

### 3.6. Sequences Obtained by PCR10

In order to obtain an even larger sequence for extended sequencing, PCR10 was developed using the forward primer of PCR6 and a new primer downstream of the reverse primer of PCR5 (Table 2). PCR10 was positive for all the tissues of all four indigenous Greek black pigs (Figure 2A), including pig 3. The amplicons from the liver and spleen of pig 3 and pig 4 were sequenced by the nanopore method and were compared with the PERV-C reference gene (Figure S4). There were significant differences, indicating that different proviruses had been amplified. When these sequences were analyzed using nBLAST, the sequences from Greek pig 3 corresponded to PERV-B and those from Greek pig 4 corresponded to PERV-A, indicating that the primers amplified non-PERV-C proviruses. PCR10 was also positive for the German slaughterhouse pig 3; the sequence of the amplicon was also identified as PERV-A. Therefore, a comparison of the primers from PCR1, PCR4, and PCR5, and the primers and probe from the real-time PCR with the corresponding binding sites in the sequences was not conclusive (Figure S4). Interestingly, PCR10 was negative for an Auckland Island pig and positive for PK15 cells (Figure 2E).

### 3.7. Screening for PERV-A/C

**Figure 4:**
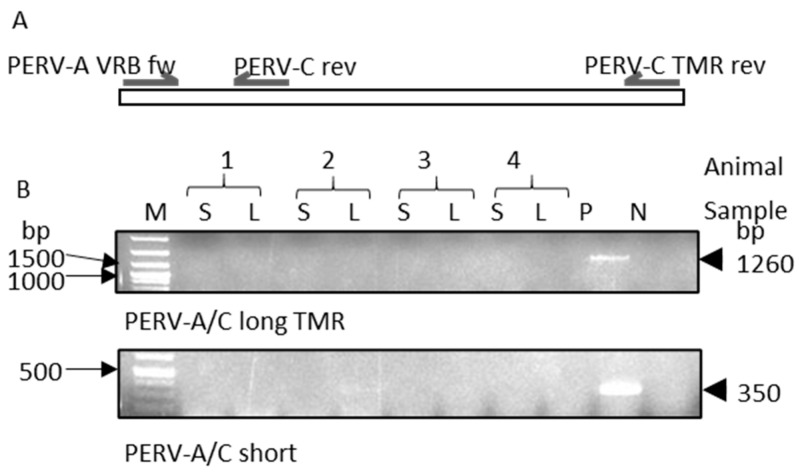
(**A**) Schematic presentation of the location of the primers in the *env* gene used to detect PERV-A/C recombinants. (**B**) Results of the PCR screening for PERV-A/C recombinants; in the upper part, the PCR results usingthe primer pair PERV-A VRB fw and PERV-C TMR rev (1260 bp amplicon) are shown, and in the lower part, the PCR results usingthe primers PERV-A VRB fw and PERV-C rev (350 bp amplicon) are shown. DNA from spleen (S) and liver (L) from four indigenous Greek black pigs from farm 1 was analyzed; in the upper part, a 1 kb plus ladder marker (M) is shown, and in the lower part, a 100 bp plus ladder is shown. DNA from 293 cells infected with a PERV-A/C released from PBMCs from a Göttingen minipig [38] was used as positive control (P); negative control (N) was water. bp, base pairs.

To detect PERV-A/C recombinants, primer pairs were used that had been previously validated for the identification of different types of recombinants [7,37,38,39,40]. Despite comprehensive screening with these primer pairs, no PERV-A/C sequences could be detected in the genome of the indigenous Greek black pigs (Figure 4).

## 4. Discussion

To perform successful xenotransplantations, the donor pigs must carry several genetic modifications that prevent rejection, and no porcine virus should be transmitted to the recipient. This requires appropriate detection methods. In this study, new PCR assays were established and applied together with already existing PCR assays [1,12,15] to screen for PERV-C proviruses in indigenous Greek black pigs, German landrace pigs and Auckland Island pigs. A total of 10 PCR assays and one real-time PCR assay were analyzed for their reliability in detecting PERV-C. Among all the analyzed assays, PCR1 and PCR4 consistently provided reliable results and should be used in future screenings for PERV-C-negative pigs. Although PERV-C does not infect human cells, it can recombine with the ubiquitous PERV-A present in all pig genomes. These PERV-A/C recombinants are able to infect human cells and replicate to higher titers compared to the paternal PERV-A [8,9]. Previously, 11 of 21 indigenous Greek black pigs in four different farms in Greece were found to carry PERV-C proviruses using PCR4 [22]. This confirms that not all pigs carry PERV-C proviruses, as reported in various previous publications. For example, 113 of 348 (30%) Chinese miniature pigs [41], 176 of 181 (97%) German transgenic and non-transgenic pigs [32], and 25 of 98 (25.5%) US farm animals [11] were found to carry PERV-C. The absence of PERV-C in several animals correlates with the fact that PERV-C is phylogenetically younger than PERV-A and PERV-B, which are present in all pigs [2,3,4].

Three of the four indigenous Greek black pigs in farm 1 were found to be PERV-C-positive using both PCR1 and PCR4 [22]. Nine out of the ten German slaughterhouse pigs were found to be positive for the virus using these PCRs, while the porcine cell line PK15 was negative for the virus, as expected. Most importantly, all the Auckland Island pigs from the CiMM in Munich were confirmed to be PERV-C-negative. In previous studies, Auckland Island pigs in New Zealand were screened for PERV-C, revealing that 27 out of 32 animals tested positive for PERV-C [30]. When we screened a different group of Auckland Island pigs, 8 out of 14 animals were found to be PERV-C-negative [21]. We selected PERV-C-negative animals, and cells from these animals were used in Germany to generate piglets through somatic cell nuclear transfer (SCNT) with surrogate mothers. The resulting piglets were screened by us [21] and confirmed to be PERV-C-negative. In this study, additional screening was conducted using the most reliable tests. The Auckland Island pigs, which have since been genetically modified for use as donor animals in a clinical trial in Germany, were also found to be PERV-C-negative.

The sequencing of the amplicon produced by PCR8 using Sanger sequencing showed that the sequence found in Greek pigs 2 and 4 from farm 1 had only very few mutations in the forward primer binding sequence and none in the reverse primer binding sequence when compared to the PERV-C reference sequence AM229312 [33]. Since the Sanger sequencing of the amplicons obtained by PCR8 using the DNA from pig 3 was not successful, possibly due to the presence of amplicons generated from different proviruses in the genome, all the amplicons were sequenced using the nanopore method. This sequencing showed insertions in the PERV sequences of animal 3. This is in agreement with the finding that the amplicons obtained when performing PCR8 using DNA from animal 3 were larger compared with the amplicons of the other three pigs. As a matter of fact, three putative insertions were found when the amplicons of PCR8 were sequenced and compared. These insertions disrupted the primer binding sites of PCR1, PCR4, and PCR5, explaining the negative results. The sequence “TGGCCATGGGAGATTGGCAACAGCGGGTACAAAAAGAT”, which is PERV-A-specific, is absent in the PERV-C sequence of animals 1 and 4, suggesting it may be a sequence derived from PERV-A that is inserted in the PERV-C sequence in animal 3. However, the sequence analysis of the amplicon from Greek pig 3 indicated that the entire sequence corresponded to PERV-A. Therefore, the so-called insertions are not insertions into PERV-C. Insertions of shorter and larger PERV-A sequences into PERV-C sequences had been observed when recombinant PERV-A/C isolated from peripheral blood mononuclear cells (PBMCs) was analyzed [38,39]; however, such PERV-A/C sequences were never found in the germ line, but only in somatic cells, indicating that the recombinants are the result of recombination and de novo integration [6].

The result of PCR8 is of particular interest. The forward primer of PCR8 is identical to that of PCR6, and the reverse primer of PCR8 is identical to that of PCR5. PCR5 and PCR6 did not produce an amplicon when DNA from Auckland Island pigs, PK15, and Greek animal 3 was tested. However, PCR5 and PCR6 were positive only with DNA from Greek animals 1, 2, and 4, which also tested positive with other PERV-C-specific PCR assays, such as PCR1 and PCR4 (Figure 2).

The real-time PCR produced equivocal results (Table 2). While the real-time PCR was negative for Auckland Island pigs, it was unexpectedly positive for Greek animal 3, which had tested negative for PERV-C using PCR1, PCR4, PCR5, and PCR6.

Of interest is the fact that PCR6 yielded a positive result when analyzing DNA from the spleen of animal 1, but not from the liver of the same animal. The negative result with liver DNA suggests that the sequence at one or both primer binding sites is either absent or mutated. This finding indicates that different proviruses are integrated into the DNA of the spleen and liver, demonstrating tissue-specific variation in a single animal.

We are confident that the methods identified as reliable are indeed robust. In this manuscript, we screened four Indigenous Greek black pigs and four Auckland Island pigs (as no additional samples were available at the time), as well as 10 German slaughterhouse pigs. However, in previous studies, we screened a total of 21 indigenous Greek black pigs [22] and 14 Auckland Island pigs [21] using the most reliable PCR tests. The samples analyzed included liver and spleen tissues, and for the Auckland Island pigs, PBMCs and cell lines were also examined. Notably, since PERVs are endogenous retroviruses, their presence should theoretically be consistent across all tissues. However, this is not the case for the copy number, which can vary depending on the tissue type [16,17,18,19].

Following the selection of PCR1 and PCR4, which have demonstrated the reliable detection of PERV-C, both methods have to be validated in accordance with the guidelines outlined in the European Pharmacopoeia and the International Council for Harmonisation (ICH) Guideline. The lower limit of detection (LOD) or detection limit (DL) will need to be established. Additionally, ongoing monitoring of the methods’ performance during routine use should be implemented.

However, the fact that a proviral sequence was classified as PERV-C according to the results of PCR1 and PCR4 does not automatically mean that this sequence corresponds to a replication-competent PERV-C or that this sequence is able to recombine with PERV-A resulting in a PERV-A/C replicating at higher titers.

For practical reasons, in order to perform safe xenotransplantations, it is important to prevent recombination between PERV-A and PERV-C leading to high-titer PERV-A/C recombinants. To achieve this, PERV-C should not be present in the genome of the donor pig. PERV-C-negative animals exist and can be identified using the PCR methods described here. Determining the presence of PERV-C proviruses using genomic sequencing technologies will be difficult due to the large number of highly related PERV sequences; highly repetitive sequences are recalcitrant to the present methods of sequencing, as shown also in the case of human endogenous retroviruses [42]. Furthermore, PERVs are active in the living pigs and the copy number of PERV proviruses depends on the pig breed, the age of the pig, and the organ tested [16,17,18,19,20,21].

The methods characterized here are required and sufficient to identify PERV-C-negative pigs for xenotransplantation to prevent recombination with PERV-A. However, we have to consider that, in addition to recombination with PERV-A, resulting in PERV-A/C recombinants, there are also other mechanisms able to transform the ecotropic PERV-C into a virus that can infect human cells. Gemeniano et al. [43] have shown that the C-terminal proline-rich region (PRR) in the envelope protein region is required in addition to the putative variable region A (VRA) and VRB for binding to cells. They showed that mutations in the PRR allowed the infection of human cells. They also showed that the cellular receptor molecule binding to PERV-C with the mutated C-terminal region of the surface envelope protein is different from the receptor molecule used when PERV-A binds and infects human cells.

There are reports demonstrating the inactivation of all PERV proviruses in the genome of a cell line [44] and the generation of piglets with inactivated PERV proviruses [45] using gene-editing techniques, primarily CRISPR/Cas9. A previous attempt to inactivate all PERVs in a pig cell line by gene editing using zinc finger nuclease (ZFN) failed [46]. However, as no PERV transmission has been observed in preclinical or clinical trials to date, it remains unclear whether this strategy is necessary. No PERV transmission was observed in numerous preclinical trials involving hundreds of non-human primates [5,47]. These trials include heterotopic heart and kidney transplantations into baboons [48], orthotopic heart transplantations into baboons [34], the transplantation of islet cells from pigs expressing INSLEA29Y into marmosets [49], the transplantation of islet cells into cynomolgus monkeys using a macrodevice [50], and the transplantation of encapsulated islet cells from Large White × Yorkshire × Landrace pigs [14] and Auckland Island pigs [27] into cynomolgus monkeys. Additionally, several clinical trials involving over 200 patients have been conducted, including islet cell transplantation for diabetes treatment, ex vivo perfusion using pig spleens and livers, and neuronal cell transplantation (for reviews, see [5,51]). In all cases, no PERV transmission was detected. More recently, two clinical trials in New Zealand and Argentina transplanted islet cells from Auckland Island pigs to treat diabetes, with no evidence of PERV transmission [28,29]. Likewise, no PERV transmission was observed in the first patients to receive a pig heart transplant [52].

The existence of pigs with inactivated PERVs throughout their entire genome, produced using CRISPR/Cas technology, raises the question of whether selecting PERV-C-negative animals is necessary. Several arguments support this approach:

1. To date, PERV has not infected recipients in preclinical and clinical xenotransplantations (see above), even when PERV-C-positive animals were used as donors.
2. Although CRISPR/Cas technology is highly specific, the risk of unintended off-target modifications in the DNA is not yet fully understood.
3. Producing a large number of CRISPR/Cas-treated animals with inactivated PERVs presents challenges, as this requires cloning, which is inefficient and may negatively impact the expression of introduced human transgenes.
  a. The cloning efficiency is relatively low. In the first reported study, 37 PERV-inactivated piglets were produced from 17 sows, but only 15 piglets survived [45].
  b. Cloning by somatic cell nuclear transfer is known to induce epigenetic variability, such as variable DNA methylation levels [53,54,55], which may affect the expression of protective transgenes that are introduced into the genome of these pigs.
4. Pig PK15 cells treated with PERV-specific CRISPR/Cas still release viral particles, although these particles are thought to be non-infectious [56]. These particles contain viral genomic RNA with an inactivated reverse transcriptase sequence. However, it cannot be excluded that these particles might enter human cells, as they carry functional envelope proteins. While the inactivated reverse transcriptase prevents viral RNA from being transcribed into DNA, human cells express reverse transcriptase from LINE sequences [57] or human endogenous retroviruses (HERVs) [58]. Consequently, the possibility cannot be ruled out that these human reverse transcriptases could potentially rescue PERVs, facilitating reverse transcription and integration.

These points highlight the complexities and potential risks involved, reinforcing the use of PERV-C-negative animals, even in the presence of CRISPR/Cas technology.

On the other hand, CRISPR/Cas technology targeting the polymerase gene allowed for the inactivation of all 62 PERV sequences in PK15 cells [44] as well as all 25 copies in embryonic cells used for the generation of newborn pigs [45], producing healthy piglets with inactivated PERVs. The porcine donor pigs were engineered to carry 69 genomic edits, eliminating glycan antigens, overexpressing human transgenes, and inactivating porcine endogenous retroviruses. The kidneys of these pigs kept nephrectomized cynomolgus macaques alive for up to 2 years in a study representing an important milestone towards the clinical translation of kidney xenotransplantation [59]. However, 40% (six recipients) were lost within the first month, with a median survival of 9 days in that group. This raises the question of whether SCNT cloning—a technology used to produce all donor animals—may have induced epigenetic variability, contributing to the differences in survival [60]. The survival time of the organs from the animals treated with CRISPR/Cas was comparable to that of donor animals without PERV inactivation. A male patient received a kidney from such a donor pig. He was able to leave the hospital and survived for two months. His death was attributed to heart disease, while the transplanted kidney remained fully functional [61,62].

## 5. Conclusions

In conclusion, screening pigs for the presence of PERV-C is easy but requires some precautions. Here, we showed that PCR1 and PCR4 consistently provided reliable results, and that these two assays should be used to screen for PERV-C in order to select PERV-C-negative animals for use in xenotransplantation. Using these two methods, we showed that one indigenous Greek black pig, all Auckland Island pigs, one German slaughterhouse pig, and the PK15 cells were PERV-C-negative. Most importantly, all the Auckland Island pigs that were genetically modified in Germany for use in clinical trials were PERV-C-negative.

## Figures and Tables

**Table 1:** Origin and characterization of the used pig materials.

Pigs, Pig Cells	Origin	Characterization
Indigenous Greek black pigs	Farm 1 located near Drama, North Greece [22]	These animals had been screened for different porcine viruses using real-time PCR (PCMV/PRV, PCV2, PCV3, PCV4, PLHV-1, PLHV-2, and PLHV-3), as well as real-time RT-PCR (HEV genotype 3), using liver and spleen tissues from 4 animals.
Auckland Island pigs	Prof. Eckhard Wolf and Dr. Barbara Keßler, Chair for Molecular Animal Breeding and Biotechnology and CiMM, Munich	Three female animals born in June 2023; they are the F1 generation of the animals born in April 2019 and were obtained by somatic cell nuclear transfer (SCNT) using PERV-C-negative kidney cells [21]. PBMCs were isolated from blood samples by gradient centrifugation, as described in [32].
German slaughterhouse pigs	Slaughterhouse near Berlin	Liver and spleen tissues from 10 animals aged 6 months.
Porcine kidney cell line PK15	Leibniz Institute DSMZ German Collection of Microorganisms and Cell lines, Braunschweig, Germany (ACC 640)	Using droplet digital PCR (ddPCR), 55 (52.5–60.1) PERV copies were detected in PK15 cells using porcine GAPDH as a reference gene, or 37 (35.0–39.1) copies using porcine beta actin (ACTB) as a reference [17].

**Table 3:** Duplex real-time PCR for the detection of PERV-C and GAPDH in indigenous Greek black pigs from farm 1, Auckland Island pigs, and German slaughterhouse pigs.

	Mean ct		260/280 nm Values ^a^
	PERV-C	pGAPDH	
Indigenous Greek black pigs, farm 1			
Pig 1 spleen	25.07	19.16	1.85
liver	26.38	19.86	1.89
Pig 2 spleen	21.06	19.14	1.90
liver	21.47	19.92	1.80
Pig 3 spleen	26.92	19.93	1.87
liver	26.23	19.98	1.82
Pig 4 spleen	21.29	19.92	1.91
liver	20.70	19.20	1.86
Positive control	19.25	19.93	n.t.
Auckland Island pigs			
13947	n.d.	19.53	1.81
13980	n.d.	19.03	1.89
13983	n.d.	19.19	1.90
Positive control	21.39	20.99	n.t.
German slaughterhouse pigs			
Pig 1 spleen	24.57	18.52	1.83
liver	28.66	20.12	1.91
Pig 2 spleen	21.67	17.68	1.80
liver	22.00	18.30	1.83
Pig 3 spleen	26.43	17.51	1.92
liver	31.05	20.08	1.84
Pig 4 spleen	25.11	18.20	1.86
liver	26.46	19.24	1.90
Pig 5 spleen	25.78	19.02	1.87
liver	28.90	20.00	1.93
Pig 6 spleen	26.36	19.39	1.89
liver	26.19	19.97	1.90
Pig 7 spleen	25.78	19.22	1.81
liver	27.42	20.17	1.82
Pig 8 spleen	23.49	19.59	1.85
liver	23.05	20.10	1.92
Pig 9 spleen	29.40	19.06	1.84
liver	31.68	19.84	1.93
Pig 10 spleen	27.12	19.12	1.87
liver	33.57	20.25	1.91
Positive control	22.03	20.10	n.t.
PK15	24.40	18.89	n.t.

^a^ indicates the purity of the DNA; n.d., not detected; n.t., not tested.

**Table 4:** Comparison of the sequences of the primers and probes used for the PCRs and real-time PCRs with the sequences found in the amplicons obtained by PCR8 using DNA from indigenous Greek black pigs 3 and 4.

PCR	Fwd/Rev			
1	fwd	Primer	CTGACCTGGATTAGAACTGG	
		Pig 3	disrupted	PCR8
		Pig 4	CTGACCTGGATTAGAACTGG	PCR8
1	rev	Primer	CCAGGACCATCCTCTAACAT	
		Pig 3	disrupted	PCR8
		Pig 4	CCAGGACCATCCTCTAACAT	PCR8
4	fwd	Primer	GATTAGAACTGGAAGCCCCAAGTGCTCT	
		Pig 3	disrupted	PCR8
		Pig 4	GATTAGAACTGGAAGCCCCAAGTGCTCT	PCR8
	rev	Primer	ACCATCCTCTAACATAACTTCTGGATCAGA	
		Pig 3	disrupted	PCR8
		Pig 4	ACCATCCTCTAACATAACTTCTGGATCAGA	PCR8
5	fwd	Primer	CTATTCGCCTCAAAATAAACCAG	
		Pig 3	CTATTCGCCTCA**G**AATA**G**A**AACT**CAG	PCR8
		Pig 4	CTATTCGCCTCAAAATAAACCAG	PCR8
	rev	Primer	CATAGAGACCAATGCACATG	
6	fwd	Primer	CCAGGACCACCAAATAATGG	
		Pig 3	not available	PCR8
		Pig 4	not available	PCR8
	rev	Primer	ACTAAAATGGGGGCAAAACTT	
		Pig 3	A**T**TAAAA**CA**GGGGCGAAACTT	PCR8
		Pig 4	ACTAAAATGGGGGCAAAACTT	PCR8
Real-time PCR	fwd	Primer	CCCCAACCCAAGGACCAG	
		Pig 3	C**T**CCAA**T**CCAAG**A**ACC**GA**	PCR8
		Pig 4	CCCCAACCCAAGGACCAG	PCR8
	probe	Probe	CTCTAACATAACTTCTGGATCAGACCC	
		Pig 3	**T**T**AC**AA**T**A**C**AAC**C**TCTGGATCAGTCCC	PCR8
		Pig 4	CTCTAACATAACTTCTGGATCAGACCC	PCR8
	rev	Primer	ACTAAAATGGGGGCAAAACTT	
		Pig 3	A**T**TAAAA**CA**GGGGC**G**AAACTT	PCR8
		Pig 4	ACTAAAATGGGGGCAAAACTT	PCR8
7	fwd corresponds to fwd primer of PCR5			
	rev corresponds to the rev primer of the real-time PCR			
8	fwd corresponds to fwd primer of PCR6			
	rev corresponds to the rev primer of PCR5			
9	fwd corresponds to the fwd primer of the real-time PCR			
	rev corresponds to the reverse primer of PCR5			

fwd, forward primer; rev, reverse primer; mutations in comparison to the primer. sequence are marked bold and grey.

## Data Availability

All data generated or analyzed during this study are included in this published article and its Supplementary Materials.

## Supplementary Materials

The following supporting information can be downloaded at https://www.mdpi.com/article/10.3390/v17020164/s1: Figure S1: Schematic presentation of the viral genomic RNA of PERV, of the integrated PERV provirus, and localization of the region where the primer pairs and the probe bind in the receptor binding site of the envelope, env, gene of PERV. LTR, long terminal repeat; R, redundant; U3, unique region 3; U5, unique region 5; gag, group-specific antigen; pol, polymerase; env, envelope. The primers are located between variable region A (VRA), variable region B (VRB), and proline-rich region (PRR) of the receptor binding site. Figure S2: Sequence alignment between the Sanger sequence of an amplicon from PCR8 of spleen DNA of animal 4 (upper line) and the PERV-C reference gene AM229312 [33] (lower line). The primer binding sites and the probe of the different PCR methods are indicated. fwd, forward primer; rev, reverse primer. Sequence 1 to 671, matches: 643; mismatches marked in red: 23; gaps: 24. Figure S3: Sequence alignment of the nanopore sequence of an amplicon from PCR8 of spleen DNA of slaughterhouse pig 3 (SP3, upper line) and the porcine endogenous retrovirus A gag-pol polyprotein and env protein genes and complete cds (AY099323.1) [36] (lower line). Figure S4: Sequence alignment of the nanopore sequence of the amplicons from PCR10 of DNA of animal 3 (p3), animal 4 (p4), and the PERV-C reference genome AM229312 [33]. Sequences of the proviruses in liver and spleen were identical. The primer binding sites and the probes of the different PCR methods are indicated. fwd, forward primer; rev, reverse primer. CLUSTAL OMEGA (1.2.4) multiple sequence alignment was used. Table S1: Overview of the PCR and real-time PCR tests conducted.
