# Supplementary material for: Reliable Polymerase Chain Reaction Methods for Screening for Porcine Endogenous Retroviruses-C (PERV-C) in Pigs
Source: Viruses. 2025 Jan 24;17(2):164. doi: 10.3390/v17020164 (PMC11860680; doi:10.3390/v17020164)
Supplement: Supplementary file 1 [file viruses-17-00164-s001.zip › viruses-3407740-supplementary.pdf]

Supplementary Materials

Figure S1

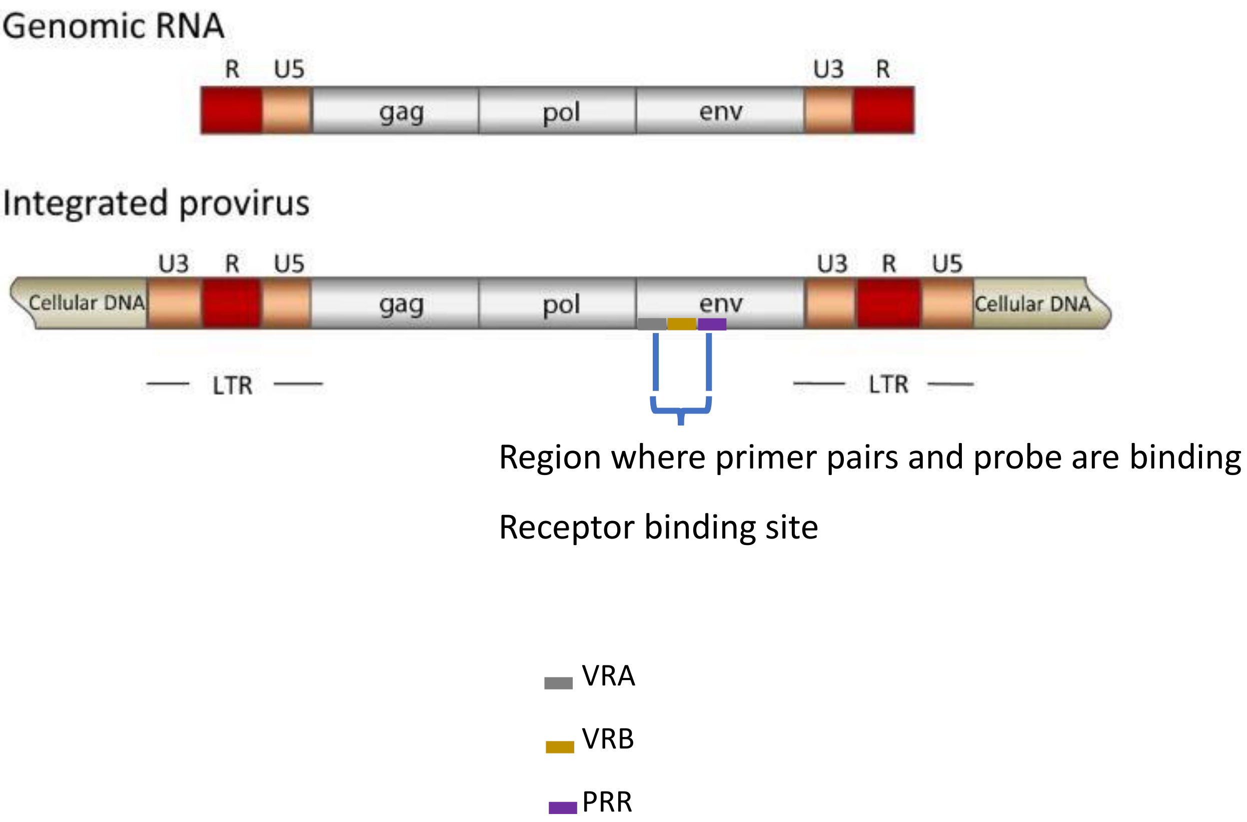

Figure S2
Figure S1

```
PCR6fwd
                                *             *             *             *             *             *             *             *
  1>~CCAG-G-A-----C---C-----A-CC-A-A-ATAATGGAAAACATTGCGGAAATCCCAGAGATTTCTTTTGTAAACAATGGAACTGTGTAACCTCTAA>80
688< |||| | |     |   |     | || | | ||||||#####|###||||||||#|||||||||||||||#|||||||||||||||||||||||||||
688<GCCAGAGCAATNTGCCTTCCCCCGACCCAACAAATAATGCCCCCCCGGGCGGAAATACCAGAGATTTCTTTTATAAACAATGGAACTGTGTAACCTCTAA<589
                                                                                                         PCR1fwd
                                                                                                         PCR4fwd
             *             *             *             *             *             *             *             *             *             *
 81>TGATGGATATTGGAAATGGCCAACCTCTCAGCAGGATAGGGTAAGTTTTTCTTATGTCAACACCTATACCAGCTCTGGACAATTTAATTACCTGACCTGG>180
588<|||||#||#|||||||||#|||##||||||||||||||||||||||||#|||||#||||#||||||||||||||||||||||||||||||||||||||
588<TGATGAATGTTGGAAATGACCAGGCTCTCAGCAGGATAGGGTAAGTTTGTCTTAGGTCAGCACCTATACCAGCTCTGGACAATTTAATTACCTGACCTG-<490

             *             *             *             *             *             *             *             *             *             *
181>ATTAGAACTGGAAGCCCCAAGTGCTCTCCTTCAGACCTAGATTACCTAAAAATAAGTTTCACTGAGAAAGGAAAACAAGAAAATATCCTAAAATGGGTAA>280
489<||||||||||||||||||||||||||||||||||||||||||||||||||||||||||||||||||||||||||||||||||||||||||||||||||||
489<ATTAGAACTGGAAGCCCCAAGTGCTCTCCTTCAGACCTAGATTACCTAAAAATAAGTTTCACTGAGAAAGGAAAACAAGAAAATATCCTAAAATGGGTAA<390

                                                            PCR5fwd
             *             *             *             *             *             *             *             *             *             *
281>ATGGTATGTCTTGGGGAATGGTATATTATGGAGGCTCGGGTAAACAACCAGGCTCCATTCTAACTATTCGCCTCAAAATAAACCAGCTGGAGCCTCCAAT>380
389<||||||||||||||||||||||||||||||||||||||||||||||||||||||||||||||||||||||||||||||||||||||||||||||||||||
389<ATGGTATGTCTTGGGGAATGGTATATTATGGAGGCTCGGGTAAACAACCAGGCTCCATTCTAACTATTCGCCTCAAAATAAACCAGCTGGAGCCTCCAAT<290

                                                    PCR4rev
                                               PCR1rev
             *             *             *             *             *             *             *             *             *             *
381>GGCTATAGGACCAAATACGGTCTTGACGGGTCAAAGACCCCCAACCCAAGGACCAGGACCATCCTCTAACATAACTTCTGGATCAGACCCCACTGAGTCT>480
289<||||||||||||||||||||||||||||||||||||||||||||||||||||||||||||||||||||||||||||||||||||||||||||||||||||
289<GGCTATAGGACCAAATACGGTCTTGACGGGTCAAAGACCCCCAACCCAAGGACCAGGACCATCCTCTAACATAACTTCTGGATCAGACCCCACTGAGTCT<190

     PCR6rev
             *             *             *             *             *             *             *             *             *             *
481>AACAGCACGACTAAAATGGGGGCAAAACTTTTTAGCCTCATCCAGGGAGCTTTTCAAGCTCTTAACTCCACGACTCCAGAGGCTACCTCTTCTTGTTGGC>580
189<|#|||||||||||||||||||||||||||||||||||||||||||||||||||||||||||||||||||||||||||||||||||||||||||||||||
189<AGCAGCACGACTAAAATGGGGGCAAAACTTTTTAGCCTCATCCAGGGAGCTTTTCAAGCTCTTAACTCCACGACTCCAGAGGCTACCTCTTCTTGTTGG-<91

v
                                                                                     PCR5rev
             *             *             *             *             *             *             *             *             *
581>TATGCTTGGCTTCGGGCCCACCTTACTATGAAGGAATGGCTAGAAGAGGGAAATTCAATGTGACAAAAGAACATAGAGACCAAT-G-CACATG>671
 90<|||||||#||||#||||||||||||||||||||||||||||||||||||||||||||||||||||||||||||||||||||| ||| | ##||
 90<TATGCTTAGCTTTGGGCCCACCTTACTATGAAGGAATGGCTAGAAGAGGGAAATTCAATGTGACAAAAGAACATAGAGAC-AATGGGGGCA~~<1
```

Figure S3

```
SP3     1      AATATTGTGGAAATCCTCAGGATTTCTTTTGCAAGCAATGGAGCTGCGTAACTTCTAATG  60
               ||||||||||||||||||||||||||||||||||||||||||||||||||||||||||||
PERV-A 371     AATATTGTGGAAATCCTCAGGATTTCTTTTGCAAGCAATGGAGCTGCGTAACTTCTAATG  430

SP3     61     ATGGGAATTGGAAATGGCCAGTCTCTCAGCAAGACAGAGTAAGTTACTCTTTTGTTAACA  120
               ||||||||||||||||||||||||||||||||||||||||||||||||||||||||||||
PERV-A 431     ATGGGAATTGGAAATGGCCAGTCTCTCAGCAAGACAGAGTAAGTTACTCTTTTGTTAACA  490

SP3     121    ATCCTACCAGTTATAATCAATTTAATTATGGCCATGGGAGATGGAAAGATTGGCAACAGC  180
               ||||||||||||||||||||||||||||||||||||||||||||||||||||||||||||
PERV-A 491     ATCCTACCAGTTATAATCAATTTAATTATGGCCATGGGAGATGGAAAGATTGGCAACAGC  550

SP3     181    GGGTACAAAAAGATGTACGAAATAAGCAAATAAGCTGTCATTCGTTAGACCTAGATTACT  240
               ||||||||||||||||||||||||||||||||||||||||||||||||||||||||||||
PERV-A 551     GGGTACAAAAAGATGTACGAAATAAGCAAATAAGCTGTCATTCGTTAGACCTAGATTACT  610

SP3     241    TAAAAATAAGTTTCACTGAAAAAGGAAAACAAGAAAATATTCAAAAGTGGGTAAATGGTA  300
               ||||||||||||||||||||||||||||||||||||||||||||||||||||||||||||
PERV-A 611     TAAAAATAAGTTTCACTGAAAAAGGAAAACAAGAAAATATTCAAAAGTGGGTAAATGGTA  670

SP3     301    TGTCTTGGGGAATAGTGTACTATGGAGGCTCTGGGAGAAAGAAAGGATCTGTTCTGACTA  360
               ||||||||||||||||||||||||||||||||||||||||||||||||||||||||||||
PERV-A 671     TGTCTTGGGGAATAGTGTACTATGGAGGCTCTGGGAGAAAGAAAGGATCTGTTCTGACTA  730

SP3     361    TTCGCCTCAGAATAGAAACTCAGATGGAACCTCCGGTTGCTATAGGACCAAATAAGGGTT  420
               ||||||||||||||||||||||||||||||||||||||||||||||||||||||||||||
PERV-A 731     TTCGCCTCAGAATAGAAACTCAGATGGAACCTCCGGTTGCTATAGGACCAAATAAGGGTT  790

SP3     421    TGGCCGAACAAGGACCTCCAATCCAAGAACAGAGGCCATCTCCTAACCCCTCTGATTACA  480
               ||||||||||||||||||||||||||||||||||||||||||||||||||||||||||||
PERV-A 791     TGGCCGAACAAGGACCTCCAATCCAAGAACAGAGGCCATCTCCTAACCCCTCTGATTACA  850

SP3     481    ATACAACCTCTGGATCAGTCCCCACTGAGCCTAACATCACTATTAAAACAGGGGCGAAAC  540
               ||||||||||||||||||||||||||||||||||||||||||||||||||||||||||||
PERV-A 851     ATACAACCTCTGGATCAGTCCCCACTGAGCCTAACATCACTATTAAAACAGGGGCGAAAC  910

SP3     541    TTTTTAGCCTCATCCAGGGAGCTTTTCAAGCTCTTAACTCCACGACTCCAGAGGCTACCT  600
               ||||||||||||||||||||||||||||||||||||||||||||||||||||||||||||
PERV-A 911     TTTTTAGCCTCATCCAGGGAGCTTTTCAAGCTCTTAACTCCACGACTCCAGAGGCTACCT  970

SP3     601    CTTCTTGTTGGCTTTGCTTAGCTTCGGGCCCACCTTACTATGAGGGAATGGCTAGAGGAG  660
               ||||||||||||||||||||||||||||||||||||||||||||||||||||||||||||
PERV-A 971     CTTCTTGTTGGCTTTGCTTAGCTTCGGGCCCACCTTACTATGAGGGAATGGCTAGAGGAG  1030

SP3     661    GGAAATTCAATGTGACAA  678
               ||||||||||||||||||
PERV-A 1031    GGAAATTCAATGTGACAA  1048
```

Figure S4

```
                PCR6fwd
p3spleen             ----------------------AACTTTGCGGGGACCCCAGGGATTTCTTTTGTAGGAAA    38
p3liver              ---------------------------TGCGGGGACCCCAGGGATTTCTTTTGTAGGAAA    33
PERV-C_AM229312      CCAGGACCACCAAATAATGGAAAACATTGCGGAAATCCCAGAGATTTCTTTTGTAAACAA    60
p4spleen             --------------------AAAATATTGTGGAAATCCTCAGGATTTCTTTTGCAAGCAA    40
p4liver              ---------------------GAATATTGTGGAAATCCTCAGGATTTCTTTTGCAAGCAA    39
                                                ** **  * **    *********** *   **

p3spleen             TGGAGCTGTGTTACCTTTAACGATGGGGATTGGAAATGGCCAACCTCCCAACAAGATTGG    98
p3liver              TGGAGCTGTGTTACCTTTAACGATGGGGATTGGAAATGGCCAACCTCCCAACAAGATTGG    93
PERV-C_AM229312      TGGAACTGTGTAACCTCTAATGATGGATATTGGAAATGGCCAACCTCTCAGCAGGATAGG    120
p4spleen             TGGAGCTGCGTAACTTCTAATGATGGGAATTGGAAATGGCCAGTCTCTCAGCAAGACAGA    100
p4liver              TGGAGCTGCGTAACTTCTAATGATGGGAATTGGAAATGGCCAGTCTCTCAGCAAGACAGA    99
                     **** *** ** ** * *** *****  **************  *** ** ** **  *

                                                                      PCR4fwd
                                                                  PCR1fwd
p3spleen             GTGAGCTTCTCCTTTATTAACTCTTTCAAAGAACTTTGGGGGTCCCGAAGAGA------T    152
p3liver              GTGAGCTTCTCCTTTATTAACTCTTTCAAAGAACTTTGGGGGTCCCGAAGAGA------T    147
PERV-C_AM229312      GTAAGTTTTTCTTATGTCAACACCTATACCAGCTCTGGACAATTTAATTACCTGACCTGG    180
p4spleen             GTAAGTTACTCTTTTGTTAACAATCCTACCAGTTATAATCAATTTAATTATGGCCATGGG    160
p4liver              GTAAGTTACTCTTTTGTTAACAATCCTACCAGTTATAATCAATTTAATTATGGCCATGGG    159
                     ** ** *  ** * * * ***      *       *      *

p3spleen             ATAGAGGGGGAAGATTCCTTATTAGGTTCATTTGATGGTCGCAAACAAAACACAGACTGT    212
p3liver              ATAGAGGGGGAAGATTCCTTATTAGGTTCATTTGATGGTCGCAAACAAAACACAGACTGT    207
PERV-C_AM229312      ATTAG------------------------------------AACTGGAAGCCCCAAGTGC    204
p4spleen             AGATGGAAAGATTGGCAACAGCGGGTACAAAAAGATGTACGAAATAAGCAAATAAGCTGT    220
p4liver              AGATGGAAAGATTGGCAACAGCGGGTACAAAAAGATGTACGAAATAAGCAAATAAGCTGT    219
                     *                                         *              **

p3spleen             TCCCCGTCAGACCTAGATTATCTAAAAATAAGTTTCACTGAAAAAGGAAAACAGGAAAAT    272
p3liver              TCCCCGTCAGACCTAGATTATCTAAAAATAAGTTTCACTGAAAAAGGAAAACAGGAAAAT    267
PERV-C_AM229312      TCTCCTTCAGACCTAGATTACCTAAAAATAAGTTTCACTGAGAAAGGAAAACAAGAAAAT    264
p4spleen             CATTCGTTAGACCTAGATTACTTAAAAATAAGTTTCACTGAAAAAGGAAAACAAGAAAAT    280
p4liver              CATTCGTTAGACCTAGATTACTTAAAAATAAGTTTCACTGAAAAAGGAAAACAAGAAAAT    279
                         * * ************  ******************* *********** ******

p3spleen             ATTCAAAAGTGGATAAATGGTATGAACTGGGGAATAGTTTTTTTATAAATATGGAGGG--    330
p3liver              ATTCAAAAGTGGATAAATGGTATGAACTGGGGAATAGTTTTTTTATAAATATGGAGGG--    325
PERV-C_AM229312      ATCCTAAAATGGGTAAATGGTATGTCTTGGGGAATGGTATATTATGGAGGCTCGGGTAAA    324
p4spleen             ATTCAAAAGTGGGTAAATGGTATGTCTTGGGGAATAGTGTACTATGGAGGCTCTGGGAGA    340
p4liver              ATTCAAAAGTGGGTAAATGGTATGTCTTGGGGAATAGTGTACTATGGAGGCTCTGGGAGA    339
                     ** * *** *** ***********   ******** ** *  *    *   *   *

                              PCR5fwd
p3spleen             GGAGCAGGGTCCACTTTAACCATTCGCCTTAGGATAGAGACGTGGACGGAACCCCCTGTG    390
p3liver              GGAGCAGGGTCCACTTTAACCATTCGCCTTAGGATAGAGACGTGGACGGAACCCCCTGTG    385
PERV-C_AM229312      CAACCAGGCTCCATTCTAACTATTCGCCTCAAAATAAACC---AGCTGGAGCCTCCAATG    381
p4spleen             AAGAAAGGATCTGTTCTGACTATTCGCCTCAGAATAGAAACTCAGATGGAACCTCCGGTT    400
p4liver              AAGAAAGGATCTGTTCTGACTATTCGCCTCAGAATAGAAACTCAGATGGAACCTCCGGTT    399
                          *** **   * * ** ******** *  *** *      *  *** ** **  *

                                          Real-timefwd
p3spleen             GCAGTGGGACCCGACAAAGTACTGACTGAACAGGGGCCCCCGGCCCTGGAGCCACCGCAT    450
p3liver              GCAGTGGGACCCGACAAAGTACTGACTGAACAGGGGCCCCCGGCCCTGGAGCCACCGCAT    445
PERV-C_AM229312      GCTATAGGACCAAATACGGTCTTGACGGGTCAAAGACCCCCAACCCAAGGACCAGGACCA    441
p4spleen             GCTATAGGACCAAATAAGGGTTTGGCCGAACAAGGACCTCCAATCCAAGAACAGAGGCCA    460
p4liver              GCTATAGGACCAAATAAGGGTTTGGCCGAACAAGGACCTCCAATCCAAGAACAGAGGCCA    459
                     **  * *****  * *  *   ** * *  **  * ** **   **  *  *     *

                                   Real-timeprobe
p3spleen             AACTTGCCGGTGCCCCAATTAACCTCGCTGCGGCCTGACATAACACAGCCGCCTGGCAAC    510
p3liver              AACTTGCCGGTGCCCCAATTAACCTCGCTGCGGCCTGACATAACACAGCCGCCTGGCAAC    505
PERV-C_AM229312      -----------------------------------TCC----------------TCTAAC    450
p4spleen             -----------------------------------TCTCCTAACCCCT-CTGATTACAAT    484
p4liver              -----------------------------------TCTCCTAACCCCT-CTGATTACAAT    483
                                                        *                     **
                                                                   Real-timerev,
                                                                   PCR6rev
p3spleen             GGTACCACTGGATTGATTCCTACCAACACGCCTAGAAACTCCCCCAGTGTTCCTGTTAAG    570
p3liver              GGTACCACTGGATTGATTCCTACCAACACGCCTAGAAACTCCCCCAGTGTTCCTGTTAAG    565
PERV-C_AM229312      ATAACTTCTGGAT---------------CAGACCCCACTGAGTCTAACAGCACGACTAAA    495
p4spleen             ACAACCTCTGGAT---------------CAGTCCCCACTGAGCCTAACATCACTATTAAA    529
p4liver              ACAACCTCTGGAT---------------CAGTCCCCACTGAGCCTAACATCACTATTAAA    528
                        **  ******               *       *      * *      *   ***

p3spleen             ACAGGACAGAGACTCTTCAGTCTCATCCAGGGAGCTTTCCAAGCCATCAACTCCACCGAC    630
p3liver              ACAGGACAGAGACTCTTCAGTCTCATCCAGGGAGCTTTCCAAGCCATCAACTCCACCGAC    625
PERV-C_AM229312      ATGGGGGCAAAACTTTTTAGCCTCATCCAGGGAGCTTTTCAAGCTCTTAACTCCACGACT    555
p4spleen             ACAGGGGCGAAACTTTTTAGCCTCATCCAGGGAGCTTTTCAAGCTCTTAACTCCACGACT    589
p4liver              ACAGGGGCGAAACTTTTTAGCCTCATCCAGGGAGCTTTTCAAGCTCTTAACTCCACGACT    588
                     *  **    * *** ** ** ***************** *****  * ********

p3spleen             CCTGATGCCACTTCCTCTTGTTGGCTTTGTCTATCCTCAGGGCCTCCTTATTATGAGGGA    690
p3liver              CCTGATGCCACTTCCTCTTGTTGGCTTTGTCTATCCTCAGGGCCTCCTTATTATGAGGGA    685
PERV-C_AM229312      CCAGAGGCTACCTCTTCTTGTTGGCTATGCTTGGCTTCGGGCCCACCTTACTATGAAGGA    615
p4spleen             CCAGAGGCTACCTCTTCTTGTTGGCTTTGCTTAGCTTCGGGCCCACCTTACTATGAGGGA    649
p4liver              CCAGAGGCTACCTCTTCTTGTTGGCTTTGCTTAGCTTCGGGCCCACCTTACTATGAGGGA    648
                     ** ** ** ** ** *********** **  *  * ** ** ** ***** ***** ***

                                     PCR5rev
p3spleen             ATGGCTAGAGAAGGAAAATTCAATGTGACCAAAAAACATAGAGATCGATGTACACGGGTA    750
p3liver              ATGGCTAGAGAAGGAAAATTCAATGTGACCAAAAAACATAGAGATCGATGTACACGGGTA    745
PERV-C_AM229312      ATGGCTAGAAGAGGGAAATTCAATGTGACAAAAGAACATAGAGACCAATGCACATGGGGA    675
p4spleen             ATGGCTAGAGGAGGGAAATTCAATGTGACAAAGGAACATAGAGACCAATGTACATGGGGA    709
p4liver              ATGGCTAGAGGAGGGAAATTCAATGTGACAAAGGAACATAGAGACCAATGTACATGGGGA    708
```

Table S1. Overview of the PCR and Real-Time PCR Tests Conducted

Pig/cell line	Animal number	Organ	Real-time PCR	PCR assays							
				PCR1	PCR4	PCR5	PCR6	PCR7	PCR8	PCR9	PCR10
Indigenous Greek black pigs	1	spleen	+	+	+	-	+/-	+	+	+	+
		liver	+	+	+	-	-	+	+	+	+
	2	spleen	+	+	+	+	+	+	+	+	+
		liver	+	+	+	+	+	+	+	+	+
	3	spleen	+	-	-	-	-	+	+	+	+
		liver	+	-	-	-	-	+	+	+	+
	4	spleen	+	+	+	+	+	+	+	+	+
		liver	+	+	+	+	+	+	+	+	+
Auckland Island pigs	13947	PBMCs	-	-	-				-		
	13980	PBMCs	-	-	-				-		
	13982	PBMCs	-	-	-				-		
	10377										-
PK15			+	-	-				-		+
German slaughter-house pigs	1	spleen	+	+	+						
		liver	+	+	+						
	2	spleen	+	+	+						
		liver	+	+	+						
	3	spleen	+	-	-						
		liver	+	-	-						
	4	spleen	+	+	+						
		liver	+	+	+						
	5	spleen	+	+	+						
		liver	+	+	+						
	6	spleen	+	+	+						
		liver	+	+	+						
	7	spleen	+	+	+						
		liver	+	+	+						
	8	spleen	+	+	+						
		liver	+	+	+						
	9	spleen	+	+	+						
		liver	+	+	+						
	10	spleen	+	+	+						
		liver	+	+	+						

+ means positive amplicon in the PCR and positive ct values in the real-time PCR, - means no amplicon in the PCR and not detected on the real-time PCR, +/- means faint band in the PCR
